# Nanocellulose-Based Inks—Effect of Alginate Content on the Water Absorption of 3D Printed Constructs

**DOI:** 10.3390/bioengineering6030065

**Published:** 2019-07-30

**Authors:** Eduardo Espinosa, Daniel Filgueira, Alejandro Rodríguez, Gary Chinga-Carrasco

**Affiliations:** 1 Chemical Engineering Department, Faculty of Science, Universidad de Córdoba, Building Marie-Curie, Campus de Rabanales, 14014 Córdoba, Spain; 2 RISE PFI, Høgskoleringen 6b, 7491 Trondheim, Norway

**Keywords:** nanocellulose, 3D printing, absorption, wound dressings

## Abstract

2,2,6,6-tetramethylpyperidine-1-oxyl (TEMPO) oxidized cellulose nanofibrils (CNF) were used as ink for three-dimensional (3D) printing of porous structures with potential as wound dressings. Alginate (10, 20, 30 and 40 wt%) was incorporated into the formulation to facilitate the ionic cross-linking with calcium chloride (CaCl_2_). The effect of two different concentrations of CaCl_2_ (50 and 100 mM) was studied. The 3D printed hydrogels were freeze-dried to produce aerogels which were tested for water absorption. Scanning Electronic Microscopy (SEM) pictures demonstrated that the higher the concentration of the cross-linker the higher the definition of the printed tracks. CNF-based aerogels showed a remarkable water absorption capability. Although the incorporation of alginate and the cross-linking with CaCl_2_ led to shrinkage of the 3D printed constructs, the approach yielded suitable porous structures for water and moisture absorption. It is concluded that the 3D printed biocomposite structures developed in this study have characteristics that are promising for wound dressings devices.

## 1. Introduction

The biomedical area is constantly developing new biomaterials with optimized properties for a given application. Wound management is a particularly demanding area with constant challenges, which motivate continuous efforts to develop tailor-made materials for specific wounds, e.g., chronic and burn wounds. In such cases, foams and hydrogels can be applied to absorb exudates, provide moisture, reduce pain and limit bacterial growth [1,2]. During the last years, cellulose nanofibrils (CNF) have appeared as a promising material for wound dressings. Importantly, CNF have several beneficial characteristics for wound dressing applications, including: absorption of large quantities of liquid, capability to form highly translucent structures with adequate mechanical properties, ability to inhibit bacterial growth and is generally cytocompatible with interesting immunogenic properties [3,4,5,6,7,8,9,10,11]. 

2,2,6,6-tetramethylpyperidine-1-oxyl (TEMPO) oxidation is one of the most commonly used methods for the production of carboxylated CNF [12]. TEMPO pre-treatment consists on the oxidation of the C6 primary hydroxyls groups of the glucose units to carboxyl groups. The higher amount of negatively charged groups increases the electrostatic repulsion between the nanofibrils, which facilitates the fibrillation and reduces the energy consumption during homogenization. Interestingly, the presence of negatively charged carboxyl groups in the CNF may provide new properties such as ionic cross-linking capability. 

TEMPO CNF-based structures have the capability to absorb large quantity of water and maintain a moistening environment suitable for wound healing [3,13]. Additionally, we have previously demonstrated that TEMPO CNF inhibits growth of *P. aeruginosa*, an opportunistic pathogen commonly occurring in infected wounds [5,14]. These characteristics suggest that TEMPO CNF could be directly used as moistening dressings for, e.g., treatment of burns. Moreover, such properties could be improved by manufacturing tailor-made porous structures by three-dimensional (3D) printing [15]. 

It is worth to mention that the hydrophilic nature of CNF, due to its large number of hydroxyl groups on their surface, and the electrical sensitivity of cellulose to water vapor, allows CNF structures to be used as moisture sensor [16,17], which was recently demonstrated for TEMPO CNF [18]. Moreover, moisture balance is especially critical in wound dressings, as excessively moist tissue can lead to maceration and insufficient moisture can lead to drying of the wound, affecting the healing process. Thus, proper wound moisture monitoring can reduce wound healing time as well as the number of dressing changes [19]. In this respect, it is also valuable to assess the moisture and water absorption capability of CNF-based materials. This will in addition provide the necessary data for clinicians to evaluate the specific wound dressing for a particular wound and wound management.

Three-dimensional (3D) printing is a layer-by-layer manufacturing process, which enables the rapid fabrication of model objects with complex structures and geometries. Depending on the technology, different raw materials (i.e., plastic, metal, ceramic, glass or biocomposites) can be used for 3D printing [20,21]. For instance, hydrogels can be 3D printed by Direct Ink Writing (DIW), which basically consists on the cold extrusion of a hydrogel through a syringe [22]. Common hydrogels used in 3D printing are biopolymers such as collagen, hyaluronic acid, chitosan or alginate [23]. A major challenge for the 3D printing of hydrogels is their collapse after 3D printing, which dramatically affects the shape fidelity of the printed structure. Due to its shear thinning behavior and rapid consolidation after deposition, CNF has excellent properties to be used in 3D printing applications [24]. Additionally, CNF can be combined with other biopolymers such as alginates to tailor the mechanical properties [25,26]. There are various types of alginates, which can be obtained from different algae and with varying composition of β-d-mannuronic acid (M) and α-l-guluronic acid (G). The composition of M- and G-blocks affects the mechanical properties of the alginates. For a good description of the effect of different alginates on the mechanical properties of CNF/alginates biocomposite gels, see Aarstad et al. [25]. 

CNF provides a rheology suitable for the extrusion process and alginate potentiates the cross-linking with divalent cations such as calcium [27]. CNF and alginates can thus be applied as inks for the controlled structuring of porous materials. Therefore, 3D printing of CNF/alginate biocomposite hydrogels is a promising pathway for the manufacturing of biobased porous structures with adequate mechanical and liquid absorption properties.

According to Boateng et al. [28], water uptake is one of the characteristics relevant for wound dressings. However, little information is available in the literature about water absorption of 3D printed constructs for wound dressing applications. Hence, in the present study, 3D printing of constructs based on CNF and varying amounts of alginate was demonstrated and the corresponding moisture and water absorption was quantified.

## 2. Materials and Methods 

### 2.1. CNF Preparation

*Pinus radiata* kraft pulp fibers (CMPC, Chile) were chemically pre-treated with (2,2,6,6-tetramethylpiperidinyl-1-oxyl (TEMPO), using 3 mmol of sodium hypochlorite (NaClO) per gram of cellulose. The kraft pulp fibers had a concentration of 1 wt% and were homogenized with a Rannie 15 type 12.56X homogenizer (operated at 1000 bar pressure). The CNF was collected after three passes through the homogenizer. The carboxyl acid content has been previously quantified to 982 ± 7.6 μmol/g for the same CNF grade used in this study [29].

### 2.2. Ink Composition 

A series of CNF-alginate compositions (inks) were prepared for 3D printing (Table 1). Alginate (PROTANAL LF 10/60, FMC corporation) and CNF were mixed using mechanical stirring until obtaining a homogeneous mixture. The amounts of alginate added were based on the dry weight of the CNF.

### 2.3. Viscosity 

The viscosities of the inks CNF, CNF_A20 and CNF_A40 were assessed using a Brookfield viscometer (Brookfield DV2TRV, John Morris Group, Sydney, Australia). The assessed volume was 20 mL at a temperature of 23 ± 1 °C and speeds of 1, 2, 6 and 10 RPM, using a spindle V-73. 

### 2.4. 3D Printing

The inks were 3D printed using a Regemat3D bioprinter (version 1.0), equipped with the Regemat3D Designer, version 1.8, Regemat3D (Granada, Spain). The target length, width and height of the 3D printed structures were 40 mm, 20 mm and 2 mm, respectively. The structures were printed directly on microscopy slides. The target width of the printed tracks was 0.41 mm. The space between the tracks was 2 mm. The flow speed was 2 mm/s, using a 0.58 mm conical nozzle. The inks were kept at room temperature (25 °C) for 24 h before printing. The 3D printed structures were cross-linked immersing the samples in calcium chloride (CaCl_2_) solution (either at concentration of 50 mM or 100 mM) for 24 h. A blank sample without cross-linking was also prepared. 

The area of the printed 3D constructs was quantified by image analysis using the ImageJ program. 

After the cross-linking the samples were flushed with distilled water to remove the excess of CaCl_2_ and freeze-dried for 24 h in a Telstar LyoQuest at −83 °C.

### 2.5. Water Absorption Capacity

The water absorption capability of the 3D printed structures was measured by immersing a pre-weighed dry sample in distilled water for 24 h and weighed at different specific times. The excess surface water was removed with filter paper before weighing. The water absorption capacity was calculated using Equation (1):

(1) $$Water\;sorption\;capacity\;\left(\%\right)=\frac{W_{t}-W_{o}}{W_{o}}\cdot 100$$

where *W_t_* is the weight of the sample at a specific time and *W_o_* is the weight of dry sample.

For measurement of moisture absorption, the prepared aerogels were placed in a climate chamber (23 °C, 90% relative humidity) during 24 h and weighed periodically. The moisture content was calculated as the difference of mass measurements at different times and the initial dry state weight using Equation (1). This analysis was carried out in triplicate and the mean value was provided.

### 2.6. SEM and Porosity

3D printed samples were prepared for scanning electron microscopy analysis (SEM, Hitachi SU3500 Scanning Electron Microscope, Hitachi High-Technologies Co., Tokyo, Japan). The freeze-dried samples were coated with a layer of gold to make the surface conductive. The equipment for gold coating was an Agar Auto Sputter Coater (Agar Scientific, Essex, UK). The images were acquired in secondary electron imaging (SEI), using 5 kV and 6 mm acceleration voltage and working distance, respectively. 

## 3. Results

**Figure 1:**
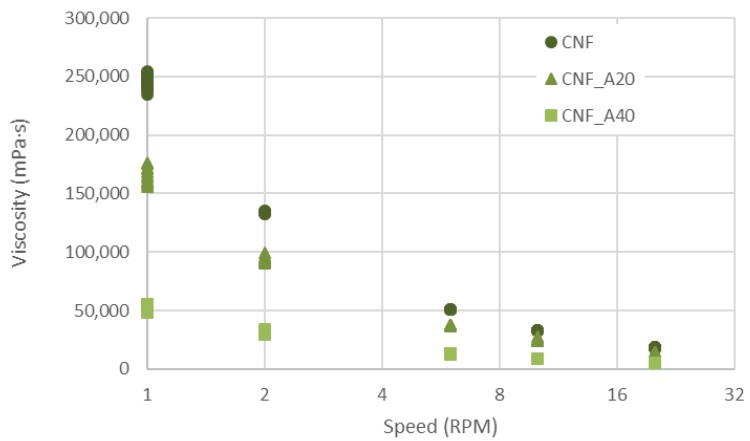
Viscosity of the inks CNF, CNF_A20 and CNF_A40. Ten single measurements are included for each speed interval and for each sample.

Figure 1 shows the viscosity of the inks CNF, CNF_A20 and CNF_A40. These compositions were used for comparison purposes. As expected, increasing the amount of alginate from 0, to 20 and 40 wt% decreases the viscosity of the inks, confirming also previous results [26]. Figure 1 exemplifies also the reduction of the viscosity as the speed increases, i.e., the inks have clear shear thinning behavior. The contribution of CNF to the rheological properties of the ink is a clear advantage for 3D printing operations. 

**Figure 2:**
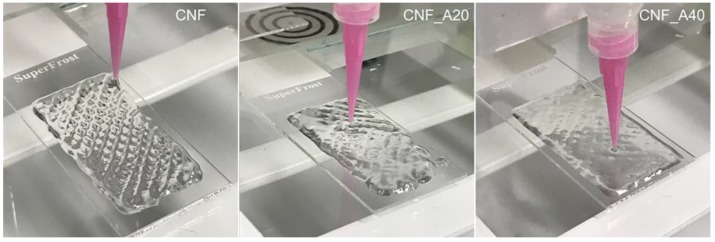
3D printed gels. Note the relatively large lateral flow of inks CNF_A20 and CNF_A40. The target dimensions of the 3D printed structures were 20 mm × 40 mm.

**Figure 3:**
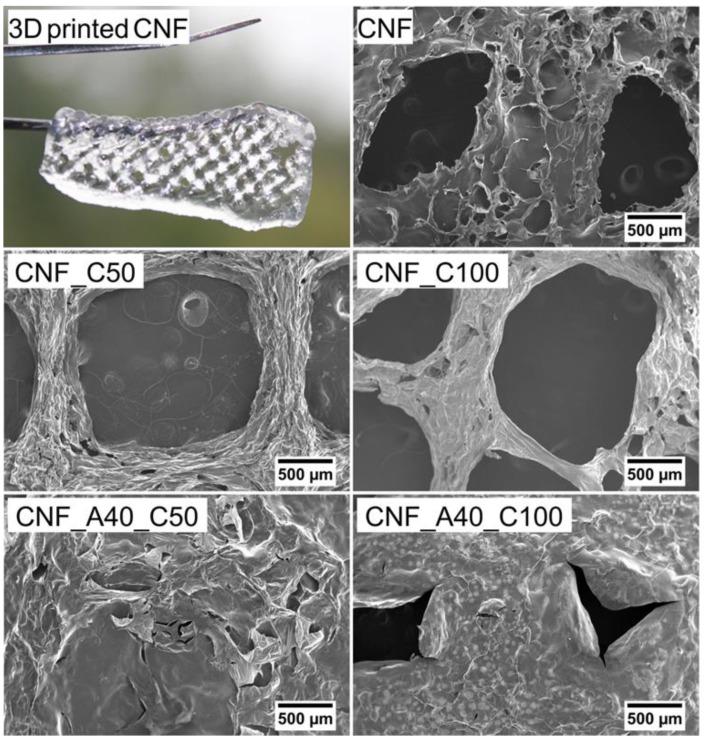
A 3D printed CNF wound dressing cross-linked with CaCl_2_ (CNF_C50), and Scanning Electron Microscope (SEM) images of a region of five freeze-dried 3D printed constructs. The target dimension of the 3D printed CNF structure (upper left) was 20 mm × 40 mm.

Structures composed of four layers (height = 2 mm) and a size of 40 × 20 mm were 3D printed (Figure 2) and freeze-dried. Morphological aspects of the aerogels made of CNF (with and without CaCl_2_ as crosslinker) and samples with 40% of alginate (with CaCl_2_ as crosslinker) were investigated by SEM images (Figure 3). The microscopy assessment reveals that an increase in the alginate content leads to a lower resolution of the 3D printed constructs. Likely, the presence of alginate in the formulation increased the lateral flow of the inks, which reduced the shape fidelity of the 3D printed objects (Figure 2 and Figure 3). The alginate applied in this study had a higher flowability compared to CNF (Figure S1: Alginate and CNF inks for 3D printing). Hence, alginate does not provide good printability and shape fidelity (Figure S2: 3D printing with alginate and CNF inks).

For the neat CNF, the spaces between the printed tracks are clearly visible (Figure 3). When the cross-linking was performed with CaCl_2_ (CNF_C50 and CNF_C100), the shrinkage of the hydrogel caused a greater definition of the printed tracks and a greater detail in the porous structure. The SEM analysis also confirmed that the inks containing alginate had a larger lateral flow, since the spaces between the tracks were not clearly visible. Such effect was lower when a higher concentration of CaCl_2_ was used. Apparently, constriction of the ink occurred during the cross-linking, as shown in sample CNF_A40_C100.

The hydroxyl, carboxyl and other polar groups found in the chemical structure of polysaccharides have the capability to form intermolecular hydrogen bonds, which have a remarkable effect on the moisture and water absorption. Moreover, the chains of polysaccharides can form networks between themselves, which keep the moisture content [30].

**Figure 4:**
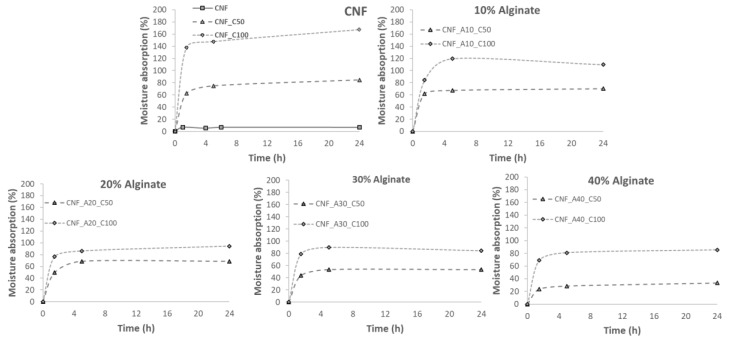
Moisture absorption curves for the different aerogels.

Figure 4 shows that neat CNF, cross-linked with CaCl_2_ had the highest moisture absorption in comparison with the inks based on the combination of CNF with alginate. The use of a higher CaCl_2_ concentration produced an increase in the moisture absorption capacity of the samples due to the hygroscopic behavior of the salt. The 3D printed structures developed in the present study showed a holding capacity of up to 165% (1.65 g of water vapor per g material) when CNF was cross-linked with CaCl_2_. Thus, the combination of both CNF and CaCl_2_ seems to be an effective material as moisture absorber. In general, the addition of alginate reduced the moisture absorption capacity of CNF. Nonetheless, the moisture absorption of the CNF-alginate structures was between 30 and 100% (0.3–1 g of water vapor per g material). 

**Figure 5:**
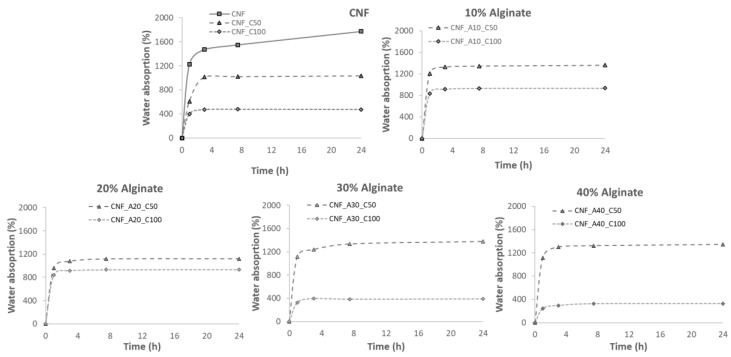
Water absorption curves for the different aerogels.

In Figure 5, the water absorption isotherms are presented for the different samples. The graphs show that the greatest capacity of water absorption is presented by neat CNF (1800% weight gain). However, the addition of alginate reduced the capacity of water absorption up to 1200% weight gain over the initial dry weight. This behavior has been reported in previous studies, where the addition of oxidized CNF improves the absorption and retention properties of alginate sponges, reaching values of 1400% of water absorbance [31]. At the same time, CNF presented better results than the use of cellulose nanocrystals. This is due to the reduction of porosity, as the internal microstructure network is modified by the interconnection of alginate with CNF. The pore network allows water molecules to pass through and fully permeate the full structure. In addition, a uniform porous structure will “lock” the water inside the structure and limit the runoff of the water [31]. It was also observed that the use of CaCl_2_ as cross-linker reduced the water absorption capacity. Hence, the higher the concentration of CaCl_2_, the lower the water absorption of neat CNF. The reduction of water absorption for the samples containing alginate was detected only for the samples cross-linked with 100 mmol CaCl_2_. It has been demonstrated that inks containing increasing alginate content led to stiffer structures after the cross-linking with Ca^2+^ [26]. During the cationic cross-linking, the ionic links between COO– groups of the oxidized CNF and the Ca^2+^ increased the cross-linking density and reduced the swelling capacity of the aerogels [32]. The cross-linking thus decreases the dimensional changes of the 3D printed structure compared with the non-cross-linked samples. This limits the deformation of the structure and thus hinders further absorption of water.

**Figure 6:**
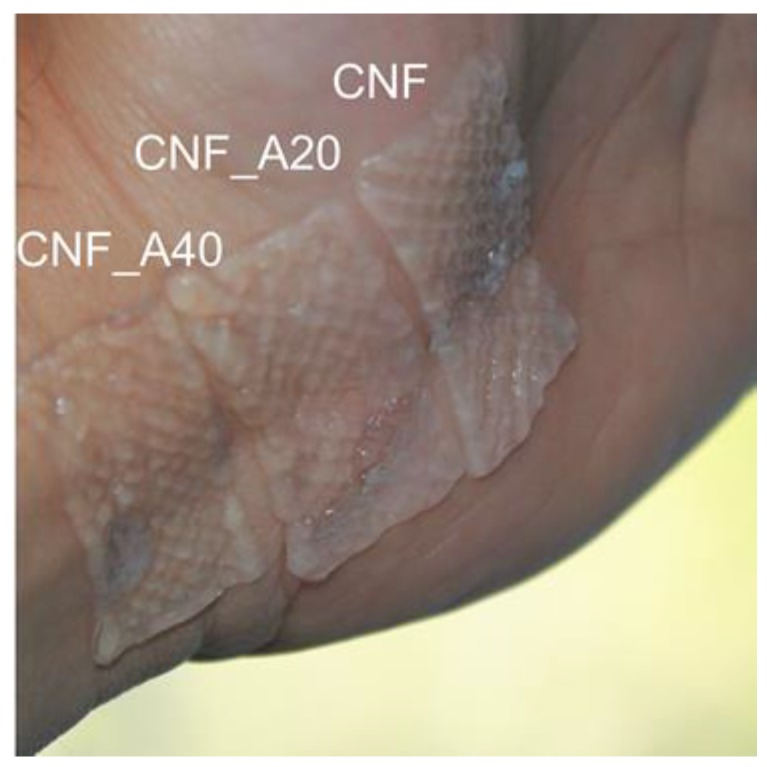
Wound dressings cross-linked with CaCl_2_ (50 mM). Note the relatively large shrinkage of the samples containing alginate. The target dimensions of the 3D printed structures were 20 mm × 40 mm.

As we have demonstrated, the wound dressings developed in this study can hold a large fraction of liquid, yet the structures were sufficiently solid to be applied as dressings, attaching and conforming easily to the surface of the skin without disintegrating (Figure 6). The quantified areas of the cross-linked dressings were 667, 612 and 519 mm^2^ for the dressings CNF, CNF_A20 and CNF_A40, respectively. Although, the alginate-containing dressings are more robust, these dressings had a larger tendency to shrink caused by the cross-linking with Ca^2+^. Based on the obtained results regarding moisture and water absorption, we propose an alginate content of 20 wt%, relative to the CNF content. This level seems to provide an adequate stability of the construct and also a good water and moisture absorption (Figure 4, Figure 5 and Figure 6). The concentration of the CNF used in this study was 1 wt%. Less shrinkage can be expected when using CNF/alginate inks with higher concentration, which may be beneficial in the design of tailor-made wound dressing devices.

It is also worth to emphasize that TEMPO CNF from the same pulp fibers applied in this study (bleached kraft softwood pulp) has been proven to be non-cytotoxic [3], with interesting dose-dependent inhibition of bacterial growth [5] and has been extensively evaluated as a potential wound dressing material [4]. Additionally, TEMPO CNF from bleached sulfite softwood pulp in combination with Ca^2+^ (as cross-linking agent) has been evaluated in in vitro and in vivo testing, demonstrating the wound healing ability of CNF hydrogels [6,7,33]. 

## 4. Conclusions

Wound dressings with a great capacity to maintain large amounts of water can be manufactured by 3D printing of CNF-based inks. The addition of alginate to the CNF and the cross-linking with CaCl_2_ consolidated remarkably the structure of the porous constructs. The highest water absorption was measured in the structures composed of neat CNF. The ionic cross-linking reduced the water absorption of CNF from roughly 1800% to 400% depending on the alginate content and Ca^2+^ cross-linking. This study demonstrates the suitability of carboxylated CNF in combination with alginate as ink for 3D printing of porous constructs for wound dressing devices.

## Figures and Tables

**Table 1:** Composition of the inks for three-dimensional (3D) printing. CaCl_2_: calcium chloride.

Series	Alginate (wt%)	CaCl_2_ (mmol)
CNF *	-	-
CNF_C50	-	50
CNF_C100	-	100
CNF_A10_C50	10	50
CNF_A20_C50	20	50
CNF_A30_C50	30	50
CNF_A40_C50	40	50
CNF_A10_C100	10	100
CNF_A20_C100	20	100
CNF_A30_C100	30	100
CNF_A40_C100	40	100

* The CNF concentration was 1 wt%.

## Supplementary Materials

The following are available online at https://www.mdpi.com/2306-5354/6/3/65/s1, Figure S1: Alginate and CNF inks for 3D printing. Note that the alginates have a higher flowability compared to the CNF. Alginates with a concentration of 1–4 wt% flow easily to the bottom of the vial (lower panel). Alginate with 8 wt% concentration has a higher viscosity as exemplified in the lower panel, when the vials are placed upside-down. Due to the high zero-shear viscosity of the CNF sample (concentration: 0.9 wt%) the material keeps its shape even when the vial is place upside-down. Figure S2: 3D printing with alginate and CNF inks. (Left) 4 wt% alginate in water. (Middle) 8% Alginate in water. (Right) 0.9% CNF in water. Note that 8 wt% alginate is required to print with some resolution, compared with CNF that prints adequately with only 0.9 wt%. Photos acquired during the printing of the 4th layer. The size of the squares is 20 mm × 20 mm.
