# Supplementary material for: Nanocellulose-Based Inks—Effect of Alginate Content on the Water Absorption of 3D Printed Constructs
Source: Bioengineering (Basel). 2019 Jul 30;6(3):65. doi: 10.3390/bioengineering6030065 (PMC6784144; doi:10.3390/bioengineering6030065)
Supplement: Supplementary file 1 [file bioengineering-06-00065-s001.pdf]

bioengineering

Supplementary Materials

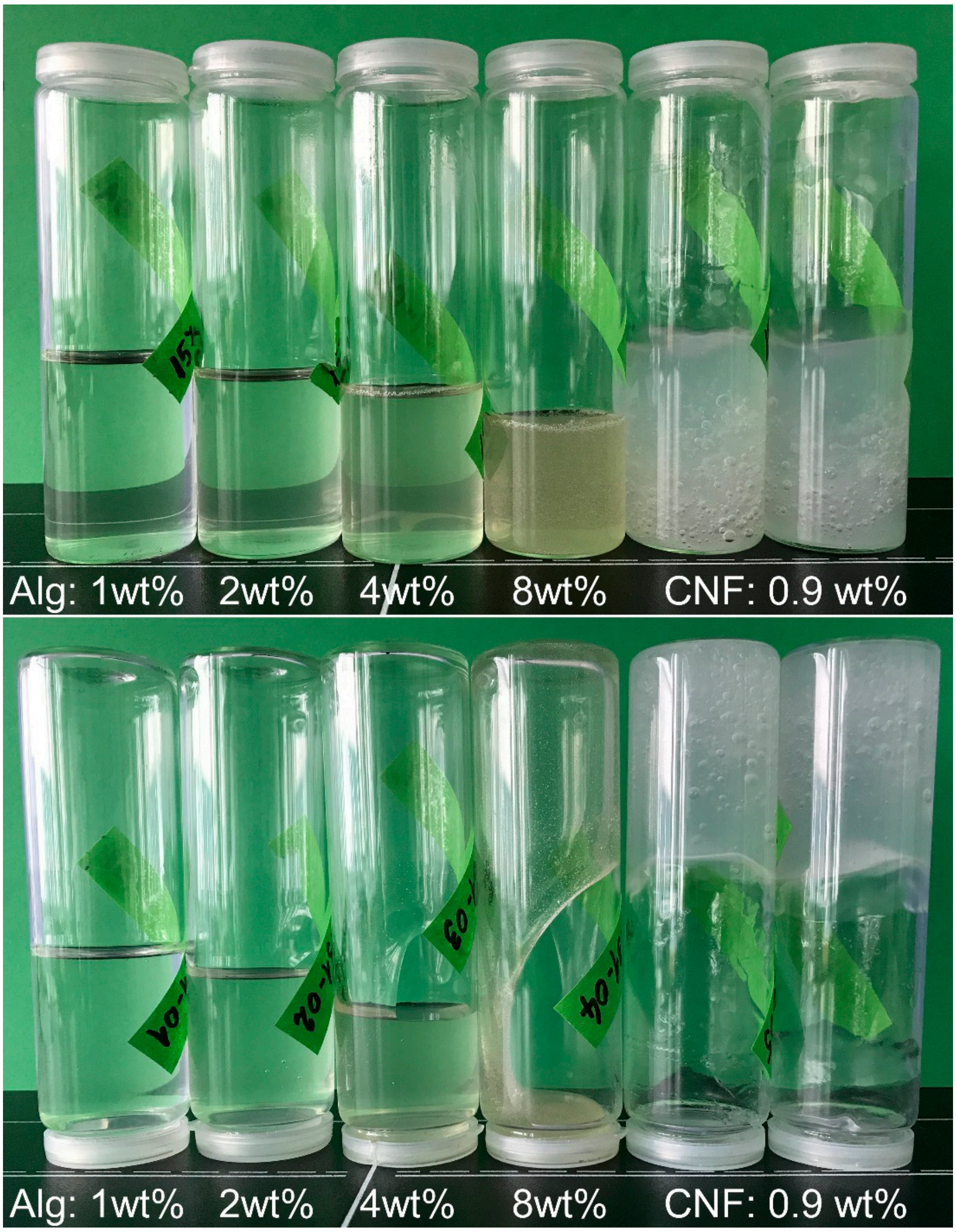

Figure S1. Alginate and CNF inks for 3D printing. Note that the alginates have a higher flowability compared to the CNF. Alginates with a concentration of 1–4 wt% flow easily to the bottom of the vial (lower panel). Alginate with 8 wt% concentration has a higher viscosity as exemplified in the lower panel, when the vials are placed upside-down. Due to the high zero-shear viscosity of the CNF sample (concentration: 0.9 wt%) the material keeps its shape even when the vial is place upside-down.

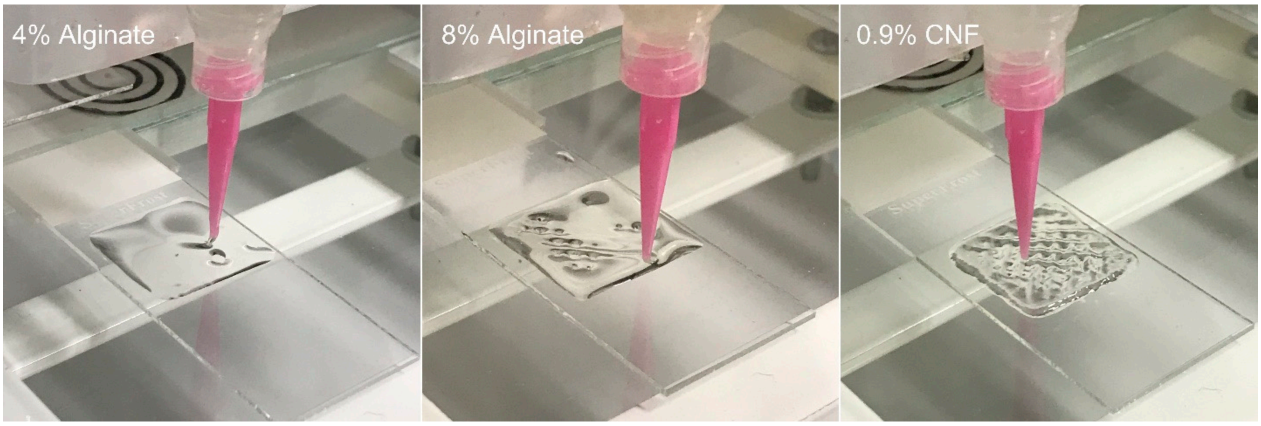

Figure S2. 3D printing with alginate and CNF inks. (**Left**) 4 wt% alginate in water. (**Middle**) 8% Alginate in water. (**Right**) 0.9% CNF in water. Note that 8 wt% alginate is required to print with some resolution, compared with CNF that prints adequately with only 0.9 wt%. Photos acquired during the printing of the 4th layer. The size of the squares is 20 mm × 20 mm.
